# The Effectiveness and Cost-Effectiveness of Web-Based and Home-Based Postnatal Psychoeducational Interventions for First-Time Mothers: Randomized Controlled Trial Protocol

**DOI:** 10.2196/resprot.9042

**Published:** 2018-01-31

**Authors:** Honggu He, Lixia Zhu, Sally Wai Chi Chan, Yap-Seng Chong, Nana Jiao, Yiong Huak Chan, Nan Luo, Shefaly Shorey

**Affiliations:** ^1^ National University of Singapore Singapore Singapore; ^2^ The University of Newcastle Newcastle Australia; ^3^ Alice Lee Centre for Nursing Studies National University of Singapore Singapore Singapore

**Keywords:** mothers, education, postpartum period, Internet

## Abstract

**Background:**

In addition to recuperating from the physical and emotional demands of childbirth, first-time mothers are met with demands of adapting to their social roles while picking up new skills to take care of their newborn. Mothers may not feel adequately prepared for parenthood if they are situated in an unsupported environment. Postnatal psychoeducational interventions have been shown to be useful and can offer a cost-effective solution for improving maternal outcomes.

**Objective:**

The objective of this study was to examine the effectiveness and cost-effectiveness of Web-based and home-based postnatal psychoeducational programs for first-time mothers on maternal outcomes.

**Methods:**

A randomized controlled three-group pre- and posttests experimental design is proposed. This study plans to recruit 204 first-time mothers on their day of discharge from a public tertiary hospital in Singapore. Eligible first-time mothers will be randomly allocated to either a Web-based psychoeducation group, a home-based psychoeducation group, or a control group receiving standard care. The outcomes include maternal parental self-efficacy, social support, psychological well-being (anxiety and postnatal depression), and cost evaluation. Data will be collected at baseline, 1 month, 3 months, and 6 months post-delivery.

**Results:**

The recruitment (n=204) commenced in October 2016 and was completed in February 2017, with 68 mothers in each group. The 6-month follow-up data collection was completed in August 2017.

**Conclusions:**

This study may identify an effective and cost-effective Web-based postnatal psychoeducational program to improve first-time mothers’ health outcomes. The provision of a widely-accessed Web-based postnatal psychoeducational program will eventually lead to more positive postnatal experiences for first-time mothers and positively influence their future birth plans.

**Trial Registration:**

International Standard Randomized Controlled Trial Number (ISRCTN): 45202278; http://www.isrctn.com/ISRCTN45202278 (Archived by WebCite at http://www.webcitation.org/6whx0pQ2F).

## Introduction

The period following a child’s birth is considered the most crucial period for mothers as they venture from their current-known reality to an unknown new reality. In addition to recuperating from the physical and emotional demands of childbirth, first-time mothers are concurrently confronted with challenges associated with the demands of adapting to their social roles as new parents [1]. In addition, they are required to pick up new skills to take care of their newborn [2]. The subsequent mastery of these necessary skills at home, without the support from health care professionals, may be challenging for some. This change in role from a nonparent, being only responsible for oneself, to having added responsibility in caring for their newborn could take a toll on first-time mothers if their expectations do not match their experiences [3]. According to the theory of maternal role attainment, a mother’s emotional outcome during this phase is very much influenced by the mother’s biopsychosocial being, family, and the environment that she is surrounded by [4]. Often, mothers are situated in an unsupported environment and may not feel adequately prepared for motherhood. This presents as a challenge physically, emotionally, and socially, and this is particularly so for first-time mothers [5]. The early postpartum phase presents a critical period where mothers prepare themselves for parenting. A mother’s negative psychological status and well-being influence the type of parenting given to their newborn in their developmental stages, consequently impairing the mother-infant relationship and impacting the child’s outcome [6].

### Postnatal Services in Singapore and Underused Antenatal Classes

To support mothers, antenatal classes and postnatal support are largely provided by the polyclinics and maternity hospitals in Singapore [7]. Antenatal classes are spread over several weeks and are usually conducted by nurses or midwives from maternity wards or private hospitals. A systematic review revealed that midwifery-led care was associated with better maternal outcomes and should be encouraged [8]. First-time mothers find that being able to communicate with a midwife regarding issues not only helped develop a trusting relationship but also helped to promote choice and control, making them feel more prepared for parenting [9]. Mothers were also satisfied with midwifery-led care [10]. However, antenatal classes usually come with a high price tag in Singapore, which mothers from lower socioeconomic backgrounds may not have access to. In contrast, such classes in certain countries in Western Europe have been offered to mothers without a fee [11]. Research also suggests that the home services provided to mothers are associated with improved emotional maternal outcomes and satisfaction [12].

### Challenges Surrounding Postnatal Period

The average length of hospital stay post delivery is approximately 24 to 72 hours for normal deliveries across all maternity hospitals in Singapore [13]. During the transitional period from hospital to home, mothers face physical challenges, including fatigue from lack of sleep. Mothers often feel a lack of support from 1 to 3 weeks after their discharge [14]. The reality of caring for their newborn only sets in after they are discharged and on their own, and this is when most learning needs are found [15]. Hence, there is a gap in the spectrum of care from the point of discharge back to their homes.

### Postnatal Depression

Postnatal depression (PND) is a depressive illness of mild to moderate severity that occurs during the first postnatal year [16]. It is one of the most common health issues that occurs among postnatal mothers globally, with most mothers experiencing PND in the first 3 months of the postnatal period [17]. In an earlier meta-analysis based on 59 studies, the prevalence of PND was approximately 13% [18]. In a recent meta-analysis of 18 studies, the overall prevalence was 20% [19], whereas another meta-analysis of 78 studies reported an overall prevalence of 3.1% [20]. The prevalence of PND in Singapore is 6.8% [21]. A local study suggested that mothers might be vulnerable to emotional distress cross-culturally regardless of their cultural postnatal traditions [22].

PND is caused by an interplay of a multitude of psychological factors, including societal expectations, social connectedness, child’s temperamental challenges, a mother’s coping strategies, and many other life stressors [23]. Physiological causes also include neurochemical imbalances in the brain [24]. If left untreated, it can persist for many years [25], leaving detrimental effects on the mother’s recovery period. Moreover, there is an association between the mother’s mental health status and maternal-child bonding, which subsequently affects the development of depression in adolescence [6]. The father and the family unit are also inevitably affected, which in turn may affect the mother’s social relationships [26,27]. These challenges are important in informing health care providers for developing a service that is essential to the promotion of a positive maternal experience. Such strategies may have an impact on future fertility rates, which is important for Singapore, a country with a rapidly aging population and a very low birth rate.

### Social Support, Maternal Self-Efficacy, and Postnatal Depression

Social support refers to interpersonal relationships that provide individuals with emotional support and self-confidence for maintaining maternal and infant psychological well-being. This form of support facilitates a mother’s adjustment into motherhood [28]. It acts as a form of buffer for mothers to mediate stress. Parenting can be psychologically stressful for first-time mothers [29], but good social support from various parties can help ease the transition phase into parenthood. The primary sources of support for a mother come mostly from their partner, other family members, and friends. However, providing support for mothers by nurses and midwives during the early postnatal period is also important, as there have been consistent evidence showing that the availability of social support contributes significantly to a person’s ability to self-regulate in the presence of stressors [30-33]. A study conducted in Singapore also underlined the significance of providing competent postnatal care to first-time mothers to improve maternal outcomes [22]. Moreover, barriers to mobilizing support were also present and these include fear of judgment and feeling like a burden [34]. Hence, having a variety of support providers can help protect mothers against the harmful psychological effects of stressors related to parenting by enhancing facilitators and reducing barriers to mobilizing support.

Another crucial component in a seamless transition to parenthood for mothers is maternal self-efficacy. Maternal self-efficacy is the belief that a mother holds of her competence in achieving a set of tasks that produces results related to parenting [35]. According to Bandura’s theory of self-efficacy, perceptions of self-efficacy come from four main sources of feedback: mastery experience, vicarious experiences, verbal persuasion, and emotional arousal [36]. For example, in the proposed study, mastery experience means the ability to strengthen self-motivation following successes in performing baby care or self-care tasks. Vicarious experiences refers to acquiring a skill through modeling from peer mothers, hence persuading herself that she, too, is able to carry out the task. Verbal persuasion is the belief that they are able to cope with baby care through encouragement from others. Emotional arousal is the psychological state of mind attributed with performing a task. Bandura’s self-efficacy theory postulates that one’s self-efficacy operates to reduce feelings of stress and depression and is crucial in determining one’s emotional reaction toward a situation.

The amount of social support received and the level of maternal self-efficacy have an impact on a mother’s emotional well-being. Inconsistent or low levels of social support have been found to be strongly associated with PND [34]. A study conducted in Canada reported the importance of social support in reducing PND [37]. Other research studies have also found that poor self-efficacy among mothers and a lack of social support are strongly associated with PND [21,38-41]. These studies support that self-efficacy, social support, and PND are associated variables, which are important in determining maternal outcomes. However, only one study has evaluated their relationships in first-time mothers, which confirmed their interrelated relationship and that all three components should be addressed [40].

### Home-Based Psychoeducation Intervention

To combat psychological stressors from parenting, psychoeducational programs aim to address knowledge deficit regarding the postnatal period and build on the participants’ strengths and resources to promote emotional coping and parenting skills development [42]. Several studies have shown that psychoeducation programs have been one of the crucial interventions to enhance maternal self-efficacy antenatally and postnatally to improve maternal outcomes [43-45]. In 2012, a preliminary study was conducted in Singapore, which reported self-efficacy improvement and PND reduction in mothers receiving a home-based psychoeducational intervention [46]. Another study exploring women’s experiences of midwifery home visits revealed that women felt vulnerable in the early postpartum period but also felt that having a personal relationship with a midwife was important [47]. However, with the shortage of midwives and nurses, a home-based intervention might not be the most accessible and cost-effective method of delivering care.

### Accessible and Cost-Effective Web-Based Psychoeducational Intervention

A Web-based intervention might be able to address the above problem by increasing accessibility and reducing costs associated with home visits without compromising the effectiveness of a psychoeducational intervention on maternal outcomes. A meta-analysis reported the effectiveness of a Web-based intervention in achieving positive outcomes for individuals [48]. Moreover, studies have shown that socioeconomic status, including low education and low income, increases the risk of first-time mothers developing depressive symptoms [49]. Therefore, examining the cost-effectiveness of a Web-based psychoeducational intervention may help to keep costs to a minimum and to make it affordable to all mothers regardless of socioeconomic status while achieving positive maternal outcomes. Moreover, in Singapore, the Home Access Program allows lower income households to be able to afford fiber broadband connectivity and a tablet at a subsidized rate, thereby increasing accessibility to resources online [50].

### Theoretical Framework

The theoretical framework adopted for this study follows Bandura’s self-efficacy theory [36], the concepts of social support including functional and structural support that can ease a smooth progression to motherhood [40,51], as well as the interrelationships between self-efficacy, social support, and emotional well-being [52-54]. The framework used in this study is similar to the one used in our preliminary study [46]. The psychoeducation intervention programs, both Web-based and home-based, are developed to promote maternal self-efficacy, social support, and mother’s psychological well-being.

### Aims

This study aims to evaluate the effectiveness and cost-effectiveness of Web-based and home-based postnatal psychoeducational programs for first-time mothers on maternal outcomes.

### Hypotheses

The hypotheses are as follows:

1. When compared with mothers in the control group, mothers in Web-based and home-based psychoeducational intervention groups will report a significantly:
  - higher level of self-efficacy,
  - higher level of social support received,
  - lower level of anxiety and depression, and
  - higher level of satisfaction with postnatal services.
2. When compared with those in the home-based psychoeducational intervention group, mothers in the Web-based psychoeducational intervention group will not report significantly poorer aforementioned maternal outcomes.
3. It is more cost-effective to provide a Web-based psychoeducational intervention than a home-based psychoeducational intervention and routine care.

## Methods

### Design

A randomized controlled three-group experimental design was used. First-time mothers (n=204) were recruited from a public hospital in Singapore. Recruited mothers were randomly assigned into any of the three groups: intervention group 1 (receiving the Web-based psychoeducational intervention plus routine care), intervention group 2 (receiving the home-based psychoeducational intervention plus routine care), and the control group (receiving routine care). Research assistant (RA2) who helped with follow-up data collection did not have any knowledge of the treatment allocation of participants, which was completed by another research assistant (RA1).

### Participants

The criteria for inclusion were first-time mothers who: (1) had full-term pregnancy, (2) were 21 years old and older, (3) were able to read and speak in English, (4) had Internet access through a computer or a smartphone, and (5) planned to reside in Singapore for the 6 months post delivery. The exclusion criteria were mothers who: (1) had identified physical or mental disorders (eg, cognitive impairment, schizophrenia, psychosis, or suicidal signs) before and during pregnancy that would hinder their ability to participate in the study, (2) had complicated assisted delivery with 4th degree perineal tear, and/or (3) gave birth to a stillborn child or a child with congenital anomalies or medical complications (eg, pathological jaundice), which required specialized attention in the hospital. A group of participants (15 from each group) were selected to participate in the process evaluation for the purpose of obtaining their opinions and comments on the Web-based, home-based psychoeducational intervention, and routine care.

### Web-Based and Home-Based Psychoeducational Intervention

The Web-based and home-based interventions were developed based on Bandura’s theory on self-efficacy [55], social support, findings from previous literature [38-40,43], and local preliminary studies [40,46,55-61]. Both interventions contain similar intervention contents but with different modes of delivery—Web-based or home visits. Table 1 shows the comparison of protocol of routine care, and Web-based and home-based postnatal psychoeducational interventions.

#### Web-Based Psychoeducational Intervention

The mothers assigned to intervention group 1 had participants receiving periodic care and a Web-based postnatal psychoeducational intervention with 1-month access. The intervention has been summarized in Table 1. The videos were developed based on current practices in local hospitals. There was a peer discussion forum where the participant could communicate with other participants, or a confidential corner for participants to ask personal questions. Expert advice was also provided by RA2 and other team members when needed. There were also thrice-weekly telephone reminders, which lasted for about 3 min each, solely as a reminder for participants to assess the website without any additional education provided.

**Table 1:** Comparison of protocol of routine care, and Web-based and home-based postnatal psychoeducational interventions.

Control group: routine care only	Intervention group 1: Web-based intervention (+ routine care)	Intervention group 2: home-based intervention (+ routine care)
Before discharge: Routine education received in the hospital	Before discharge: Access to website information, and Web-based audio and video materials	Before discharge: Provision of booklet
	Main content covered in the website, audios, and videos, which included the following: postnatal experiences; maternal self-care (including physical, emotional, and sexual health); newborn care, and social support	Main content covered in the booklet included the following: postnatal experiences; maternal self-care (including physical, emotional, and sexual health); newborn care, and social support
At 2 weeks post-delivery: Follow-up with obstetrician; examination of episiotomy or cesarean wound; wound dressing for cesarean wound; and use of Edinburgh Postnatal Depression Scale (EPDS)	1st week after discharge: Go through website information, and Web-based audio and video materials	1st week after discharge: One hour face-to-face education at home
	Peer discussion forum, and a confidential corner for personal questions and expert advice	Main content covered in the face-to-face session is the same as in the booklet
	Mothers can discuss their concerns related to self-care and newborn care; expert (RA2 and other team members) will access the website and respond to the questions raised daily	
At 6 weeks post-delivery: Follow-up with the obstetrician; advice on: breastfeeding; PAP smear; intrauterine contraceptive device upon patients’ request; and EPDS for those mothers who have defaulted the 2 weeks appointment	2nd to 4th week after discharge: Weekly telephone calls x 3; reinforce the use of website resources	2nd to 4th week after discharge: Weekly telephone calls x 3; reinforcement of the content covered during home visit; find out new challenges faced by the mothers; and provide individualized support as per mothers’ needs

#### Home-Based Psychoeducational Intervention

Participants assigned to intervention group 2 received periodic care and a home-based postnatal psychoeducational intervention. There was a 1-hour face-to-face psychoeducation via a home visit by a registered nurse (RA1). An educational booklet was also developed based on the one used in the preliminary study [61] with new evidence-based knowledge added, such as mindfulness-based practice and using cold cabbage leaves for managing breast engorgement [61], which were provided to participants before hospital discharge. The main contents in the booklet provided for the home-based intervention were identical to the one from group 1. The contents were validated by an expert panel, consisting of 5 health care professionals (2 professors in Obstetric and Gynecological Nursing, 1 senior consultant in obstetrics, 1 experienced midwife in postnatal care, and 1 postnatal psychologist). There was also a thrice-weekly telephone follow-up to answer mothers’ queries.

### Control Group

Participants assigned to the control group only received the routine care provided by the hospital.

### Sample Size Determination

In similar studies, psychosocial and educational interventions have resulted in a medium effect size on outcomes similar to this study [44,52,62]; hence, the study intervention was regarded to have a medium size effect on outcome variables. On the basis of a power analysis, to achieve a medium effect size of 0.55, using a power of 80% and a significance level of 5% (2-sided), 52 participants would be needed in each group [63]. Although a dropout rate of 30% was reported by a previous study [52] and our preliminary study [46], we anticipated a lower dropout rate of 24% in this study because of better communication channels established with participants (eg, using social media such as WhatsApp to remind participants, follow-up questionnaire conducted through a Web-based survey). Therefore, a minimum sample of 204 (52×3/0.76) participants with 68 in each group was needed. For process evaluation, a purposive sampling of 45 participants (15 from each group) with different posttest 1 self-efficacy scores (5 high, 5 moderate, and 5 low scores from each group) were invited to participate in a process evaluation interviews, or until data saturation was achieved.

### Randomization

A random sequence generator was used to generate three sets with 68 unique random integers in each set that ranged from 1 to 204 without being sorted [64]. Three sets of numbers were randomly assigned for intervention group 1, intervention group 2, or control group, respectively. The blinding of the participant was not possible because of the nature of the intervention. All 204 unique numbers indicating the group were placed in an opaque box. After assessing eligibility and obtaining informed consent, participants were assigned randomly to either of the groups by drawing a number from the box. Participants were reminded that there will be no change of groups after numbers are drawn.

### Outcome Measures and Instruments

The outcome measured included are maternal parental self-efficacy (MPSE), social support, PND, anxiety, satisfaction with postnatal care, and cost related to health care services used postnatally. The instruments used to measure the key variables included Perceived Maternal Parental Self-efficacy (PMPSE), the modified Perinatal Infant Care Social Support (PICSS-modified), Edinburgh Postnatal Depression Scale (EPDS), Anxiety subscale of the Hospital Anxiety and Depression Scale (HADS-A), Ordinal Descriptive Scale (ODS), and Questionnaire on Healthcare Service Utilization (QHSU).

#### Perceived Maternal Parental Self-Efficacy

The PMPSE is a widely used 17-item instrument that measures maternal self-efficacy in the postpartum period. Each item is rated on a four-point Likert scale. The Cronbach alpha values in a previous study and a local study were .91 [65] and .92, respectively [54].

#### Social Support

The modified PICSS includes the Functional Social Support Measuring Subscale (FSSMS) and Structural Social Support Measuring Subscale (SSSMS). The FSSMS is a 16-item subscale used to measure maternal perceived social support [57] with Cronbach alpha values of .80 in a previous study [57] and .76 in a local study [54]. The SSSMS is a 6-item subscale used to measure the structural dimension of an individual’s social network with a Cronbach alpha of .83 in a local study [54].

#### Psychological Well-Being

The EPDS and HADS-A were used to measure mothers’ psychological well-being, including PND and anxiety. The 10-item EPDS has been used to measure PND widely. The recommended cutoff for probable cases of PND is 12 or 13 [66]. The sensitivity and specificity of the EPDS were 68% to 80% and 77%, respectively, and the Cronbach alpha was .88 [67]. The HADS-A is a 7-item instrument that is most commonly used to assess anxiety symptoms [68]. It is rated on a 4-point scale of 0 to 3. The minimum score of 8 determines the presence of anxiety symptoms [69]. The sensitivity and specificity of HADS-A were 93% and 90%, respectively, when it was used for pregnant women from Nigeria [70].

#### Satisfaction With Postnatal Care

The ODS is a 6-point Likert scale for assessing mothers’ satisfaction with the postnatal care they received [46,59].

#### Health Care Service Utilization

The QHSU was used to capture costs related to health care service usage by the mothers postnatally due to maternal- or infant-related health issues at 1 month, 3 months, and 6 months post delivery. The measurement period for the study was from entry into the program until 6 months. The questionnaire was designed by health economy experts from the Singapore Clinical Research Institute.

### Process Evaluation

Semistructured interviews were conducted for exploring the information regarding the strengths, weaknesses, and perceived effects of the interventions, and the mothers’ opinions on the current routine postnatal care provided by the hospital.

### Study Procedure

Data collection commenced after the ethical approval had been obtained. The nurse in charge was contacted to determine the physical and psychological well-being of potential participants. After confirming the inclusion criteria, the participants were approached at the postnatal inpatient wards. Only after obtaining written consent would the mother be recruited into the study. This was followed by a collection of demographic data and baseline data via a self-administered questionnaire before randomization takes place. Outcomes were measured at the following time points for all participants in all three groups: (1) after delivery and before discharge (baseline data), (2) immediately after the intervention (posttest one, at the end of 1 month post delivery), (3) 2 months after the intervention (posttest 2, 3 months post delivery), and (4) 5 months after the intervention (posttest 3, 6 months post delivery).

RA1 made a visit to the hospital regularly to recruit participants and was responsible for informing all participants of their allocated group, and collection of baseline data for all participants. RA1 provided participants in the Web-based intervention group with Web access (individual username and password) for 1 month. For participants assigned to the home-based intervention group, RA1 provided the educational booklet before discharge and arranging home visits at about 5 to 10 days post delivery, as this is the most crucial period for mothers, as well as 3 telephone calls [71].

RA2 was responsible for the Web-based postnatal psychoeducational intervention (eg, providing expert advice in group discussion forums and confidential corner, and 3 reminder phone calls). RA2 was responsible for the collection of all posttest data, including process evaluation interviews.

### Data Analysis

Quantitative data will be analyzed using IBM SPSS version 24 for Windows (IBM Corp). Descriptive statistics such as mean, standard deviation, and range will be used to report continuous data, whereas frequency and percentages will be used for the nominal and ordinal data. Inferential statistics such as ANOVA will be used to analyze the differences of baseline outcomes among the three groups, whereas chi-square tests will be used to compare the sociodemographics among the three groups.

To answer the hypothesis 1, repeated measures ANCOVA, adjusted for confounding variables (eg, age, ethnicity), will be used to test the effects of both interventions on maternal self-efficacy, social support, postnatal depression, and anxiety across four time points. Univariate ANCOVA will be used to test the difference of each of the three outcomes among three groups at each posttest time point by adjusting for confounding factors (eg, baseline data, age, and ethnicity). Chi-square test or Poisson regression will be used to determine maternal satisfaction with the postnatal supportive care at each posttest time points. To answer hypothesis 2, a 95% CI of the mean difference of maternal self-efficacy scores will be calculated. If the lower boundary is more than −5, the hypothesis will be accepted. To address hypothesis 3, we will calculate the base case incremental cost-effectiveness ratios (ICER) of the two intervention groups compared with the control group. We captured the 6-month costs at three posttest time points: (1) health care system perspectives, including service providers that mothers consumed because of maternal and/or neonatal related conditions such as GP clinic, polyclinic, private clinic, hospital out-patient, hospital Accident and Emergency and (2) participant’s out-of-pocket cost of transportation fee for these visits. In addition, fixed costs (also considered health care system perspectives) such as development and implementation of Web-based intervention, delivery of home-based intervention, and training of RAs were also captured. The effectiveness of the intervention on four outcomes (namely self-efficacy, social support, depression and anxiety) will be calculated. The ICERs for each outcome will be plotted in the cost-effectiveness plane to examine the dominance and extended dominance, and then the cost-effectiveness acceptability curves will be generated to demonstrate the cost-effectiveness of the alternatives using difference willingness-to-pay thresholds [72-78].

Qualitative data from the interviews will be analyzed using thematic analysis [79-83]. The audiotaped interview data will be transcribed verbatim by RA2 concurrently with the data collection to capture nonverbal nuances. Transcribed data will be color coded first, which will then be collated to form subthemes, and eventually to form themes [84]. Two investigators will be involved in the analysis process to compare and discuss the codes, subthemes, and themes that are generated and to achieve consensus. Rigor, including credibility, transferability, dependability, and confirmability, will be considered carefully in the study process [85].

### Ethical Considerations

Ethics approval for the study was received from the relevant ethics review board before commencing the study (Ref: 2015/01189) on 25 January 2016. A participant information sheet, which provides a brief introduction to the study as well as the benefits and risks of their participation in the study was explained. Anonymity and voluntary participation was ensured. Each participant’s written consent was also obtained, including consent for audio recording during the process evaluation interview. Participants whose EPDS scores were more than 13 were referred to their obstetrician for further follow-up. A token of appreciation was provided to all participants for their time spent to participate in the study and this is a common practice in the study country.

## Results

**Figure 1:**
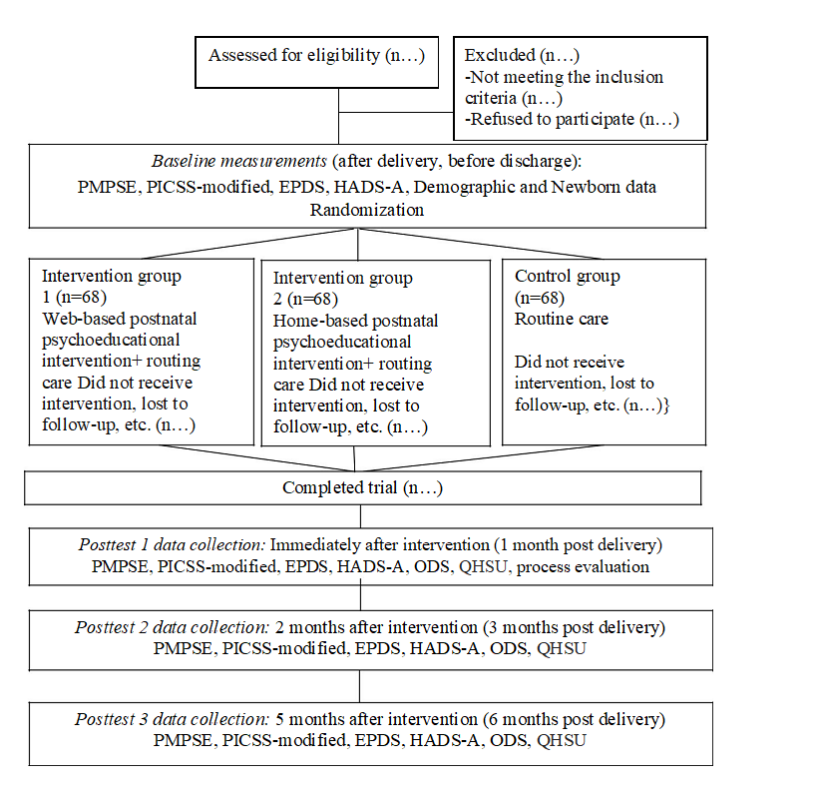
Consolidated standards of the reporting trial flowchart of the study. PMPSE: Perceived maternal parental self-efficacy; PICSS-modified: Modified Perinatal Infant Care Social Support Scale; EPDS: Edinburg Postnatal Depression Scale; HADS-A: Hospital Anxiety and Depression Scale (Anxiety Subscale); ODS: Ordinal Descriptive Scale (satisfaction with postnatal care); QHSU: Questionnaire on Healthcare Services Utilization.

The booklet for the home-based intervention group and the website for the Web-based intervention (including the audio and video files) have been developed. The recruitment of participants started in October 2016 and was completed in February 2017 with 68 mothers in each group (n=204). All follow-up data were collected by August 2017. The consolidated standards of reporting trial flowchart can be found in Figure 1. The projected timeline for completion of data entry and data analysis is around March to June 2018.

## Discussion

### Principal Findings

This proposed study is based on previous studies conducted by the same research team [22,46,59,60]. With minimal time spent from midwives and/or nurses, this study will provide empirical support and identify a clinically useful and potentially effective and cost-effective Web-based postnatal psychoeducational program in promoting maternal self-efficacy, seeking social support from health care professionals, and family members), and psychological well-being by equipping mothers with knowledge, skills, and support in the postnatal period. The Web-based psychoeducational intervention will reduce the need for face-to-face home-based interventions. If the proposed Web-based psychoeducational intervention is as effective and more cost-effective when compared with the home-based intervention, it will be introduced to the hospital policy maker and be adopted as a standard care to promote the quality of postnatal care for the new mothers and increase mothers’ satisfaction with postnatal services, without needing extra manpower to provide postnatal home visits to mothers.

The short term clinical implication of this study is such that a comprehensive and well-designed Web-based postnatal psychoeducational program will be developed for first-time mothers. Following that, an understanding will be established on the determinants of the outcome variables in improving maternal outcomes. These strategies can also be incorporated into the services provided by midwifery-led clinics.

In the long term, the practicality of both interventions will be determined. If the Web-based intervention is reported to be more practical, effective, and cost-effective as compared with the home-based intervention and the current standard routine care, the learning package adopted in the Web-based intervention can then be provided to first-time mothers to address any doubts and questions. With the implementation of the Web-based intervention, first-time mothers will then be able to effectively allocate their time and resources when it comes to the usage of emergency medical services in addressing minor issues related to maternal and newborn care. Health care professionals will also be able to effectively allocate appropriate resources when it comes to educating new mothers and providing consultations to address queries from mothers. The Web-based intervention can also be beneficial to mothers as it provides continuity of care for new mothers after their discharge from the hospital and enhances the safety of both the mother and the newborn. Empowering mothers with confidence back at home contributes to the overall well-being of mothers and newborns [46]. A positive postnatal experience could also potentially influence future birth plans and eventually contribute to an increase fertility rate in Singapore. In addition, mothers in countries other than Singapore can also benefit from the website resources.

### Limitations of the Study

One of the limitations was the inability to blind the participants from the intervention because of the nature of the interventions. This study was only conducted in a single tertiary hospital in Singapore and all participating mothers were English-speaking mothers. The results tested on mothers from the private hospitals and mothers who speak other languages might be different.

### Conclusions

This randomized controlled trial will provide empirical evidence to support the effectiveness and cost-effectiveness of the Web-based and home-based psychoeducational interventions in promoting maternal outcomes. This study also has potential benefits in reducing health care costs associated with postnatal supportive services. Future studies should include non-English-speaking mothers and mothers from other maternity hospitals in Singapore and other countries to enhance the generalizability of the study findings.

## Abbreviations

CONSORT: Consolidated Standards Of Reporting Trial
CEAC: cost-effectiveness acceptability curves
EPDS: Edinburg Postnatal Depression Scale
FSSMS: Functional Social Support Measuring Subscale
HADS-A: Hospital Anxiety and Depression Scale
ICER: incremental cost-effectiveness ratios
IUCD: intrauterine contraceptive device
MPSE: Maternal Parental Self-efficacy
NHG DSRB: National Health Group Domain Specific Review Board
ODS: Ordinal Descriptive Scale
PMPSE: Perceived Maternal Parental Self-efficacy
PND: postnatal depression
QHSU: Questionnaire on Healthcare Services Utilization
SSSMS: Structural Social Support Measuring Subscale
SPSS: Statistical Package for the Social Sciences

## Appendix

Multimedia Appendix 1 Original peer-review reports.
