# Supplementary material for: The Effectiveness and Cost-Effectiveness of Web-Based and Home-Based Postnatal Psychoeducational Interventions for First-Time Mothers: Randomized Controlled Trial Protocol
Source: JMIR Res Protoc. 2018 Jan 31;7(1):e35. doi: 10.2196/resprot.9042 (PMC5812979; doi:10.2196/resprot.9042)
Supplement: Multimedia Appendix 1 [file resprot_v7i1e35_app1.pdf]

Proposal S/N: HSRG13MAY003

Principal investigator: Dr He Hong-Gu **Institution:** NUHS

Proposal title: **The effectiveness and cost-effectiveness of web-based and home-based postnatal psychoeducational interventions for first-time mothers: A randomized controlled trial**

Please respond to reviewer comments as far as possible, making use of the box to the right below.

**Reviewer Comments**	**PI's Response**
**Feasibility**	
1. It may be rather ambitious to undertake a three-arm RCT with resources for only two years so the applicants will need to plan their time and resources very carefully.	Yes we fully agree with the reviewer that it is ambitious to undertake this study in two years. However, we will plan the time and resources very carefully as suggested by the reviewer.<br><br>Following are some of the justifications of our plans:<br><br>In the first 6 months, we will obtain the ethical approval from NHG DSRB and develop the web-based intervention concurrently. The PI has conducted more than 15 projects that needed DSRB approval and her experience will facilitate the DSRB application process for this study. We will include A/Prof. Stan Jarzabek in the team as a co-investigator (if allowed by MOH, he has already agreed). A/Prof. Jarzabek is working at the School of Computing at NUS and his expertise is in the area of mHealth, tapping the unique potentials of mobile technology to improve delivery of healthcare interventions. His expertise will be instrumental in developing the web-based intervention programme with the time frame.<br><br>In our preliminary randomised controlled two-group pre- and post-tests study, we spent 3 months to recruit 122 first-time mothers and another 3 months to complete the 3-month follow-up data collection in 2012 (Dragon year). We estimate that each month we can recruit 20-30 participants in this study; it may take about 8-10 months to recruit 204 participants. The last follow up will be 6 months after intervention, therefore we plan 14-16 months for the recruitment and data collection. The rest 2-4 months will be used to analyse the data, write the report and prepare the manuscripts.<br><br>The PI and the team have experiences on the recruitment, intervention (home-based) and data collection for the similar group of participants from the same study hospital. This will help us to conduct the proposed study.

**Reviewer Comments**	**PI's Response**
**<u>Cost-effectiveness analysis</u>**	
2. Information on method of costing the interventions is limited. The applicants should ensure they have in place a realistic approach for estimating indirect time (administration, preparation, training, supervision etc) and be clear on their method of valuation, i.e. are they using a micro-costing (bottom-up) approach?	Retrospective self-report of the time spent (by PI, each Co-I, RA) using a standardised questionnaire will be used to capture the relevant data. Each team member will keep a log book to record the relevant information (including the time spent in administration, preparation, training, supervision etc) for better estimation of the time spent at the end of the study.<br><br>We will not use a micro-costing (bottom up) approach to estimate this cost component.
3. Similarly, little information is provided on the analysis beyond the use of the ANOVA. The applicants need to consider how to deal with the fact that cost data are generally skewed – the standard approach is to use parametric tests but to test the assumptions using bootstrapping (see e.g. various papers by Barber & Thompson on the analysis of cost data).	We will report the arithmetic mean (standard deviation) and interquartile range to provide better picture of the data.<br><br>Because the distribution of cost is generally skewed, as mentioned by the reviewer, we will use a non-parametric bootstrapping method to estimate the difference in cost between treatment arms and associated 95% confidence intervals.
4. The applicants state that cost-effectiveness is the difference between unit costs for the three intervention groups, divided by the difference between outcomes. This is not the case. When more than two strategies are compared, incremental cost-effectiveness ratios are calculated using rules of dominance and extended dominance (see Johannesson & Weinstein, 1993). In addition, it would be common to present results using cost-effectiveness acceptability curves (see e.g. the papers by Fenwick et al).	Apologies for the mispresentaion of the statement.<br><br>To determine the cost-effectiveness of the two interventions, we will calculate the incremental cost-effectiveness ratios (ICER) of the two intervention groups as compared to the control group.<br><br>The ICERs will be plotted in the cost-effectiveness plane to examine the dominance and extended dominance, and then the cost-effectiveness acceptability curves (CEAC) will be generated to demonstrate the cost-effectiveness of the alternatives using different willingness-to-pay thresholds.

Reviewer Comments	PI's Response
5. Whilst it is true that "a cost-effectiveness analysis can be calculated with…a number of independent outcomes", as is being suggested here, it is unusual since it then becomes difficult to make a decision if cost-effectiveness is better on one measure and worse on another. It would be more common to select a primary measure that you hypothesise will be the most relevant and then to present the alternative measures in a cost-consequences analysis.<br>a. *Barber, J. A. & Thompson, S.G. (1998) Analysis and interpretation of cost data in randomised control trials: review of published studies. BMJ, 317,1195^2000.*<br>b. *Fenwick, E. & Byford, S. (2005) A guide to costeffectiveness acceptability curves. British Journal of Psychiatry, 187,106^108.*<br>c. *Fenwick, E.,Claxton,K. & Sculpher,M. (2001) Representing uncertainty: the role of cost-effectiveness acceptability curves. Health Economics, 10, 779-787.*<br>d. *Johannesson,M. & Weinstein,M.C. (1993) On the decision rules of cost-effectiveness analysis. Journal of Health Economics,12, 459-467.*	Just as commented by the reviewer, it is possible that the cost-effectiveness may be better on one measure and worse on another. Therefore, we will perform the cost-effectiveness for all outcomes (primary and secondary), but we will make recommendations based on the result of the primary outcome (maternal self-efficacy).

Reviewer Comments	PI's Response
**Process Evaluation**<br>6. There is a lack of clarity around the method for the process evaluation.	
○ The section on sample size for the process evaluation (p. 8) does not provide any literature to support theoretical sampling or to justify the purposive sample size of 36 mothers.<br>○ This section would benefit from improved justification for the sample and reference to supporting literature (e.g. Coyne, 1997; Guest, Bunce, & Johnson, 2006; Higginbottom, 2004).	In our preliminary study (Shorey et al., 2014), we conducted process evaluation interviews on the purposive sample of first-time mothers from the intervention group. We reached data saturation with 12 mothers. Therefore, instead of theoretical sampling, we proposed a purposive sample of 36 mothers (12 in each group). We will recruit the mothers based on post-test 1 results of their perceived self-efficacy in newborn care (4 high, 4 moderate, and 4 low scores from each group). The aim is to obtain description of mothers' opinions and comments on the postnatal care, the web-based or home-based psycheducational programmes. The proposed sample size is just a guide and recruitment will continue until the data saturation is achieved. The recommended literature will be cited in the proposal (e.g. Coyne, 1997; Guest, Bunce, & Johnson, 2006; Higginbottom, 2004).
Is the proposed qualitative inquiry a qualitative descriptive approach (Sandelowski, 2000; Sandelowski, 2010)? Qualitative descriptive methodology has been described as a discovery-oriented, naturalistic approach that stays closer to the data than more interpretive qualitative approaches such as phenomenology (Chenail, 2011).	Yes this proposed qualitative inquiry is a descriptive qualitative approach (Sandelowski, 2000; Sandelowski, 2010), which means that it is a discovery-oriented, naturalistic approach that will stay close to the data.
○ The qualitative data analysis section (p. 12) cites a paper that discusses phenomenological hermeneutical method (Reference 151), which does not appear to fit well with the method actually described in the application.	The Reference 151 (Lindseth & Norberg, 2004) will be removed from the proposal.
○ This section needs improvement, perhaps with reference to analysis relevant to health services research (e.g. Bradley, Curry & Devers, 2007).	Based on reviewers' comments, this section will be improved by incorporating the relevant literature (Bradley, Curry & Devers, 2007; Gao & Chan, 2012). In addition, the PI and the team members have experiences of conducting similar descriptive qualitative studies using thematic analysis, which have been published by various journals. This experience will help with the data analysis of the qualitative data from the process evaluation.
a. *Bradley, E. H., Curry, L. A., & Devers, K. J. (2007). Qualitative data analysis for health services research: developing taxonomy, themes, and theory. Health Services Research, 42(4), 1758-1772.*<br>b. *Chenail, R. J. (2011). How to conduct clinical qualitative research on the patient's experience. Qualitative Report, 16(4), 1173-1190.*<br>c. *Coyne, I. T. (1997). Sampling in qualitative research. Purposeful and theoretical sampling; merging or clear boundaries? Journal of Advanced Nursing, 26(3), 623-630.*<br>d. *Guest, G., Bunce, A., & Johnson, L. (2006). How many interviews are enough? An experiment with data saturation and variability. Field methods, 18(1), 59-82.*<br>e. *Higginbottom, G. M. (2004). Sampling issues in qualitative research. Nurse Researcher, 12(1), 7-19.*<br>f. *Sandelowski, M. (2000). Whatever happened to qualitative description? Research in Nursing & Health, 23(4), 334-340.*<br>g. *Sandelowski, M. (2010). What's in a name? Qualitative description revisited. Research in Nursing & Health 33(1), 77-84.*	*Shorey, S., He, H.G., Chong, Y.S. & Chan, S.W.C. (2014). Process Evaluation of postnatal psychoeducation programme on first-time mothers. Midwifery. (Submission 9 March 2014; Revision submitted 14 May 2014, YMIDW-D-14-00074.R1)*<br><br>*Gao, L.L., Chan, S. (2012). The interpersonal-psychotherapy-oriented program for Chinese pregnant women: delivery, content and personal impact. Nursing and Health Sciences, 14, 318-324.*

Reviewer Comments	PI's Response
**Other Comments**	
7. The inclusion of a component of Cognitive-Behaviour Therapy in the form of a discussion about irrational versus rational thoughts in both the web-based and booklet psycho-education may be harmful to participants. Mothers in the post-partum period are psychologically vulnerable and taking one component of a treatment paradigm and delivering it out-of-context and without the considerable psycho-education that needs to be provided before discussing cognitive distortions and cognitive techniques for replacing these with more adaptive and helpful thinking styles is potentially harmful. Additionally, these techniques are discussed with mothers for only 10 minutes. It is not feasible to do this intervention within that time frame. The researchers should consider deleting this aspect of the psycho-education protocol as it confounds the study. It is not possible to determine if the improved/unchanged/deteriorated psychological well-being will be an outcome of the psycho-education or of the attempt at cognitive therapy for postnatal depression.	The cognitive-behaviour therapy in the form of a discussion about irrational versus rational thoughts in both web-based and booklet psychoeducation will be removed from the study as suggested.
8. The recruitment of mothers to the study is obtained by accessing mother and baby medical records without the consent of the mother herself. This is not considered ethical conduct in many international countries. Consent of the mother needs to be obtained prior to perusal of medical records and mothers need to be informed that they may/may not meet the selection criteria for inclusion in the study.	In our preliminary study, we used the same approach to recruit mothers, which was approved by DSRB.<br><br>However, in order to be consistent with the practice in many other international countries, as suggested by the reviewers, we will revise the recruitment procedure. That is, we will approach all first-time mothers to obtain their verbal consents for us to access to their medical records and meanwhile they will be informed that they may/may not meet the selection criteria for inclusion in the study. Only after obtaining their verbal consents we will access to their medical records and decide whether they are eligible to be recruited.

Reviewer Comments	PI's Response
9. It seems unlikely that the researchers will be able to achieve all of the objectives of the phone contacts with Intervention group 2 within 10 minutes. The amount of time the researcher spends talking to mothers on the phone will likely impact on the level of social support mothers perceive and therefore on the results of the study. If the study is aimed at evaluating the efficacy of the intervention (web-based versus brochure based) itself, then the level of social support by the researcher needs to be controlled for.	Based on the preliminary RCT we conducted, the time for the phone call for intervention group 2 will be increased to 20-30 minutes, depending on the needs of individual mother. This is to ensure that the objectives of the phone follow up are met especially answering mothers' queries that may develop post home visits.<br><br>We don't see the need of controlling the social support by the researcher as the intervention group 1 will also receive the expert advice on their queries via the web-based forum on regular bases.
10. [Page 7 Sample size calculation] Justify if proposed sample size of 68 per group (after factor-in 30% attrition rate) using the repeated measures ANOVA tests is sufficient for comparisons of secondary outcomes (e.g. social support) and non-inferior testing of web-based vs. home-based psychoeducational intervention groups (i.e. Hypothesis 2 on page 1)	In this study, the sample size was calculated based on the primary research outcome (maternal self-efficacy). All secondary outcomes will be studied in an exploratory nature.<br><br>In addition, the sample size was not calculated based on the non-inferior hypothesis for two reasons: 1) this is only one of our secondary research questions; 2) should we calculate the sample size based on this non-inferiority study, we will need a much bigger sample size (for example, based on our primary outcome, if I use 5 as equivalence range, SD 10, mean difference 0, the minimum sample size is 112 per group, considering the 30% attrition rate, we will need 146 per group and 438 in total), which is impossible to achieve in a two-year project. Therefore, this question will also be studied in an exploratory nature.
11. [Page 7 Randomisation] The proposed randomisation method is weak, i.e. the 3rd allocation is predictable after the 1st and 2nd allocations. Blocked randomisation with random sequences of mix block sizes can ameliorate this problem.	As suggested by the reviewer, we will change the randomisation method and use blocked randomisation with random sequences of mix block sizes in this study.

Reviewer Comments	PI's Response
12. [Page 11 Data Analysis] To provide specific analysis methods for each hypothesis, primary and secondary outcomes instead of providing generic analysis methods.	All quantitative data will be analysed using IBM SPSS 22.0. Missing data will be replaced (depending upon the amount of missing data assuming <10%) for intention-to-treat analysis. Both intention-to-treat analysis and per-protocol analysis will be conducted to compare any differences between groups. Descriptive statistics such as mean, standard deviation and range for continuous data and frequency as well as percentages will be used for the nominal and ordinal data. Cronbach's alpha value will be used to examine the internal consistency of the questionnaires. Inferential statistics, such as Independent sample t-test or Analysis of Variance (ANOVA) will be used to compare the differences of outcomes between/among the demographic subgroups.<br>***For hypothesis 1:***<br>Presuming that the outcomes are normally distributed, parametric tests will be used. Repeated measures multivariate analysis of covariance (MANCOVA) adjusted for confounding variables (e.g. age, education level) will be used to test the effects of both interventions on outcomes including maternal self-efficacy, social support and postnatal depression across four time points of data collection (Triply MANCOVA). The percentage changes of the maternal self-efficacy, social support and postnatal depression scores from baseline will be calculated for repeated measures triply MANCOVA. Multivariate analysis of covariance (MANCOVA) will be used to test difference in each outcome among three groups at three post-tests separately. Chi-square test or Fisher's exact test will be used to determine maternal satisfaction with postnatal supportive care at 1 month, 3 month and 6 month postpartum.<br>***For hypothesis 2:***<br>The 95% confidence interval of the mean difference of maternal self-efficacy scores will be calculated. If the lower boundary is more than -5, the non-inferiority of the web-based intervention as compared to the home-based intervention will be accepted.<br>***For hypothesis 3:***<br>Descriptive statistics of cost, including arithmetic mean (standard deviation) and interquartile range, will be calculated. Because the distribution of cost is generally skewed, we will use a non-parametric bootstrapping method to estimate the difference in cost between treatment arms and associated 95% confidence intervals. To determine the cost-effectiveness of the two interventions, we will calculate the incremental cost-effectiveness ratios (ICER) of the two intervention groups as compared to the control group. The ICERs will be plotted in the cost-effectiveness plane to examine the dominance and extended dominance, and then the cost-effectiveness acceptability curves (CEAC) will be generated to demonstrate the cost-effectiveness of the alternatives using different willingness-to-pay thresholds.

Reviewer Comments	PI's Response
13. To complement the team by including a statistician with experience in the implementation and analysis of randomised controlled trial.	Dr. Chan Yiong Huak, an experienced statistician, has been invited to be a co-investigator of the project. However, he is overseas during this period; hence his CV will be provided in due time. He will help with the cost-effectiveness analysis and other data analysis. Dr. Chan played an important role in analysing data for our preliminary study.
**Research Team**	
14. The PI has a track record that indicates relevant experience in educational interventional studies, although less experience in randomized trial implementation.	The PI has experiences in conducting various randomised controlled trials, such as:<br>○ "The effectiveness of therapeutic play intervention on outcomes of children undergoing inpatient elective surgery: a randomized control trial" (PI), funded by NMRC NIG. The study has been completed with one paper published and three are under review. *(See references list 1-4)*<br>○ "Effectiveness of self-efficacy enhancing educational programme on outcomes of first-time mothers in Singapore: a randomized controlled trial" (Co-I), funded by Sigma Theta Tau International Upsilon Chapter Research Grant. This study was completed in 2012. Two articles reporting the finding have been published and three manuscripts are under review. *(See references list 5-9)*<br>○ "The effectiveness of educational intervention on outcomes of parents and their children who undergo inpatient elective surgery: a randomized controlled trial" (PI), funded by MOH HSR CRG. The study is on-going and the data collection will be completed in Dec. 2014. One manuscript is under review. *(See references list 10)*<br><br>Hence, the PI is confident to implement the proposed RCT with the prior experiences. In addition, the variety of expertise from other team members will ensure the successful implementation of this study:<br>○ Prof. Sally Chan: Psychoeducation intervention studies using RCTs<br>○ A/Prof. Chong Yap Seng: Clinical experience and access to participants<br>○ A/Prof. Stan Jarzabek: mHealth and web-based intervention development<br>○ Dr. Luo Nan: Cost-effectiveness analysis<br>○ Dr. Shefaly Shorey: Perinatal care experiences and preliminary psychoeducation intervention using RCT<br>○ Dr. Chan Yiong Huak: Statistical analysis

Reviewer Comments	PI's Response
15. None of the investigators have any training or experience in providing cognitive-behavioural therapy.	Professor Sally Chan has the experiences in using cognitive-behavioural therapy principles in programmes similar to this study. Please find the following references (Gao et al., 2012; Ngai et al., 2009). However, the cognitive-behaviour therapy in the form of a discussion about irrational versus rational thoughts in both web-based and booklet psychoeducation has been removed from the intervention programme as suggested in Comment 7.<br><br>*Gao, L.L., Chan, S., Sun, K. (2012). Effects of an Interpersonal-Psychotherapy-oriented childbirth education programme for Chinese first-time childbearing women: three-month follow-up. International Journal of Nursing Studies, 49, 274-281.*<br>*Ngai, F.W., Chan, S., Ip, W.Y. (2009).The effects of a childbirth psychoeducation program on learned resourcefulness, maternal role competence, and perinatal depression. International Journal of Nursing Studies, 46, 1298-1306.*

References (Manuscripts prepared from the RCTs conducted by the PI in the previous three years)

1. *He, H.G.*, Zhu, L.X., Li, H.C.W., Wang, W., Vehviläinen-Julkunen, K., & Chan, W.C.S. (2014). A randomized controlled trial of the effectiveness of a therapeutic play intervention on outcomes of children undergoing inpatient elective surgery: study protocol. Journal of Advanced Nursing, 70(2), 431–442. doi: 10.1111/jan.12234.*
2. *He, H.G.*, Zhu, L.X., Klainin-Yobas, P., Chan, W.C.S., & Wang, W. (2014). The Effectiveness of Therapeutic Play Intervention in Reducing Perioperative Anxiety, Negative Behaviors and Postoperative Pain in Children Undergoing Elective Surgery: A Systematic Review. Pain Management Nursing. (Submission 14 Nov 2013, PMN-D-13-00087)*
3. *He, H.G.*, Zhu, L.X., Chan, W.C.S., Liam, L.W.J., Li, H.C.W., Ko, S.S., Klainin-Yobas, P., & Wang, W. (2014). The effects of a therapeutic play intervention on children's perioperative anxiety and postoperative pain: a randomized controlled trial. Journal of Advanced Nursing. (Resubmitted to JAN 12 April 2014; JAN-2014-0391; Revision July 2014)*
4. *He, H.G.*, Zhu, L.X., Chan, W.C.S. Li, H.C.W., Liam, L.W.J., Ko, S.S., Wang, W., & Klainin-Yobas, P. (2014). Parents' perioperative anxiety and their perceptions of a therapeutic play intervention for their children. Journal of Advanced Nursing. (Submission 26 March 2014, JAN-2014-0324; Revision July 2014)*
5. *Shorey, S., Chan, W.C.S., Chong, Y.S., & He, H.G.* (2014). A randomized controlled trial of the effectiveness of a postnatal psychoeducation programme on outcomes of primiparas: study protocol. Journal of Advanced Nursing. doi: 10.1111/jan.12461*
6. *Shorey, S., Chan, W.C.S., Chong, Y.S., & He, H.G.* (2014). Predictors of maternal parental self-efficacy among promiparas in the early postnatal period. Western Journal of Nursing Research. Doi: 10.1177/0193945914537724*
7. *Shorey, S., Chan, S.W.C., Chong, Y. P., & He, H.G.* (2014). A randomized controlled trial of the effectiveness of a postnatal psychoeducation program on 3-month follow up outcomes of primiparas. Journal of Advanced Nursing. (JAN-2014-0366.R1; Revision 1 July 2014)*
8. *Shorey, S., He, H.G.*, Chong, Y.S. & Chan, S.W.C. (2014). Process Evaluation of postnatal psychoeducation programme on first-time mothers. Midwifery. (Revision submitted 14 May 2014, YMIDW-D-14-00074.R1)*
9. *Shorey, S., Chan, S.W.C., Chong, Y. P. & He, H.G.* (2014). The effects of a six-week postnatal psychoeducation program on self-efficacy, social support and postnatal depression among first time Singaporean mothers: A randomized controlled trial. Patient Education and Counseling. (Submitted 21 May 2014; PEC-14-434)*
10. *He, H.G.*, Zhu, L.X., Chan, W.C.S., Xiao, C.X., Klainin-Yobas, P., Wang, W., Cheng, K.F.K., & Luo, N. (2014). A randomized controlled trial of the effectiveness of educational intervention on outcomes of parents and their children undergoing inpatient elective surgery: study protocol. Journal of Advanced Nursing. (Submitted 11 April 2014; Revision submitted 14 June 2014, JAN-2014-0390.R1)*
